# CRISPR Screening: Molecular Tools for Studying Virus–Host Interactions

**DOI:** 10.3390/v13112258

**Published:** 2021-11-11

**Authors:** Vladimir Chulanov, Anastasiya Kostyusheva, Sergey Brezgin, Natalia Ponomareva, Vladimir Gegechkori, Elena Volchkova, Nikolay Pimenov, Dmitry Kostyushev

**Affiliations:** 1 National Medical Research Center of Tuberculosis and Infectious Diseases, Ministry of Health, 127994 Moscow, Russia; vladimir@chulanov.ru (V.C.); kostyusheva_ap@mail.ru (A.K.); seegez@mail.ru (S.B.); ponomareva.n.i13@yandex.ru (N.P.); n.pimenov@mail.ru (N.P.); 2 Scientific Center for Genetics and Life Sciences, Division of Biotechnology, Sirius University of Science and Technology, 354340 Sochi, Russia; 3 Department of Infectious Diseases, Sechenov University, 119991 Moscow, Russia; az@rcvh.ru; 4 Department of Pharmaceutical and Toxicological Chemistry, Sechenov University, 119991 Moscow, Russia; vgegechkori@gmail.com

**Keywords:** SARS-CoV-2, COVID-19, hepatitis, HIV, Cas12, Cas9, HAV, ZIKV

## Abstract

CRISPR/Cas is a powerful tool for studying the role of genes in viral infections. The invention of CRISPR screening technologies has made it possible to untangle complex interactions between the host and viral agents. Moreover, whole-genome and pathway-specific CRISPR screens have facilitated identification of novel drug candidates for treating viral infections. In this review, we highlight recent developments in the fields of CRISPR/Cas with a focus on the use of CRISPR screens for studying viral infections and identifying new candidate genes to aid development of antivirals.

## 1. Introduction

Viruses are obligate pathogens that use the host–cell machinery for replication. Host cells can recognize the virus and activate antiviral responses. Revealing the factors that affect viral infection can aid in discovery of new drug candidates. Using specific immune agonists that contribute to antiviral immune responses is another approach for treating infections. Studying host–cell interactions and identifying critical targets for developing new antivirals have recently become possible using new, comprehensive molecular tools such as clustered regularly interspaced short palindromic repeats (CRISPR) screens. Over the last decade, CRISPR/CRISPR-associated protein (Cas) systems have been adopted for genome editing. Cas proteins recognize the target site using single-guide RNA (sgRNA) in type II CRISPR systems and CRISPR RNA (crRNA) in type V systems (for simplicity, named gRNA in this review) only if the genomic target is followed by a protospacer adjacent motif (PAM) sequence (NGG for Cas9 protein and TTTV for Cas12a protein). Upon target site recognition, Cas proteins unwind the DNA strands, forming an R-loop structure, and cut the two strands, resulting in a DNA double-stranded break (DSB).

Using site-specific mutagenesis, a variant of Cas endonuclease with nucleolytically blunt domains has been generated, known as nucleolytically dead Cas (dCas) protein. dCas retains the ability to bind the site of interest but cannot introduce DSBs into DNA. Fusing different functional domains to dCas proteins transforms them into molecular “Swiss army knives” with a variety of functions, such as single-nucleotide editing and regulating transcription and epigenetics [1]. Activating or inhibiting target gene transcription by different CRISPR/Cas systems has been widely used to disrupt individual genes and study virus–host interactions [2]. By designing and synthesizing thousands of gRNAs targeting multiple genes of interest or all genes in the genome, it is possible to use CRISPR/Cas techniques to directly investigate the function of host factors in a large-scale format, referred to as CRISPR screens [3]. Using sets of gRNAs (gRNA libraries), CRISPR screens enable perturbation of thousands of genes simultaneously to evaluate their functions in a single experiment.

CRISPR screens are widely used to investigate complex biological processes, including virus–host interactions, in a high-throughput manner. These cutting-edge molecular tools enable whole-genome analysis of host factors, identifying and validating the effects of these factors on viral replication. This manuscript reviews the basic principles, types, and workflow of CRISPR screens. In the second part of this review, we provide a comprehensive analysis of recent discoveries made using CRISPR screens for investigating virus–host interactions and identifying new antiviral drug targets.

## 2. CRISPR/Cas-Based Molecular Tools Used for CRISPR Screens

Classical Cas9 nucleases (type II CRISPR/Cas systems) recognize a specific target and introduce DNA DSBs, which are then repaired by error-prone cellular mechanisms. Disrupting gene coding sequences by frameshift mutations enables the use of classical Cas9 nucleases for gene knockout applications (CRISPR-ko).

Cas12a enzymes (type V CRISPR/Cas systems) possess RNAse activity, so several gRNAs can be transcribed as a single transcript that is further processed by Cas12a into individual gRNAs [4]. This unique feature of Cas12a facilitates multiplex targeting and makes Cas12a particularly suitable for combinatorial CRISPR screening.

Instead of inactivating two nucleolytic domains, as dCas9 proteins do, leaving a single functional nucleolytic domain repurposes Cas9 from introducing DSBs to nicking a single DNA strand. These modified Cas9 proteins are named Cas9 nickases (nCas9). Fusing dCas9 or nCas9 proteins with different functional domains endows them with new properties. Based on the functional domain used, dCas9/nCas9 systems can be adapted for a wide range of biological applications (reviewed in [1]), including:

- DNA base editing (cytidine deaminases APOBEC3A [5], rAPOBEC1 [6], AID [7])
- Gene repression via CRISPR-interference (CRISPRi): dCas9 fused with KRAB [8,9,10], EZH2 [10], KRAB-MeCP [11], DNMT3A [12], DNMT3L [13]
- DNA methylation or de-methylation (DNMT3A [13,14] or TET1 [15]) for modulating gene expression
- Gene activation via CRISPR activation (CRISPRa) (VPR [16,17], SAM [18], SunTag-VP64 [19] and others)

Base editing can be achieved using nCas9 fused with a DNA deaminase domain (APOBEC3A, rAPOBEC1, AID, or their improved variants) [20]. Cytidine base editors mediate deamination of target cytidine nucleotides, resulting in G→A/C→T mutations [21], and can be used for DSB-independent gene knockout.

dCas9 protein fused to transcriptional repressors is used for CRISPRi (gene repression). The KRAB domain is a eukaryotic transcriptional repressor commonly used for CRISPRi. dCas9-KRAB systems suppress gene transcription by recruiting epigenome remodeling cellular enzymes that add inhibitory epigenetic modifications to target loci [22]. The disadvantage of dCas9-KRAB inhibitors is variable efficacy, depending on loci and gRNAs, as well as the transient nature of gene inhibition [22]. The combined system dCas9-KRAB-MeCP2 demonstrates higher inhibitory activity than dCas9-KRAB alone, as MeCP2 attracts additional repressors, thus prolonging the effects of transcriptional inhibition and enhancing efficiency [11]. Adding DNA methyltransferase domains DNMT3A-3L to dCas9-KRAB (DNMT3A-3L-dCas9-KRAB; CRISPR-off) potentiates the inhibitory activity and duration of the epigenetic memory, so that repression of gene transcription persists even after dCas9 systems are released and degraded [23].

To activate genes, dCas9 has been fused to different activation domains. One of the most powerful activation tools is the three-component complex of transcriptional activators VP64-p65-Rta (VPR) characterized by substantially higher gene activation efficiency than first-generation systems. Another effective activation system is the synergistic activation mediators (SAM) system consisting of fused dCas9-VP64 protein and modified gRNA containing two MS2 RNA aptamers [18]. MS2 aptamers in gRNA can recruit up to four copies of aptamer-binding MCP proteins linked to p65-HSF1 activation domains. In the SunTag activation system [12], dCas9 is linked to an array of short GCN4 peptide repeats; VP64 activation domains are coupled to anti-GCN4-scFv fragments. Using antibody–antigen interaction, dCas9-GCN4 mobilizes up to 10 scFv-VP64 domains to the regulatory site. Current advances in the CRISPR toolbox have been described in detail elsewhere [1,24,25,26].

## 3. Types of CRISPR Screens

### 3.1. Pooled and Arrayed CRISPR Screens

Screening protocols can be run in two formats: arrayed screens or pooled screens. Arrayed screens are usually conducted in 96-well plates with 1 gRNA per each well, while in pooled screens, cells are transduced with a gRNA mix. The desired phenotype is selected, and the mutation that caused the desired phenotype is identified using next-generation sequencing (NGS) [27]. CRISPR screens for viral applications are typically performed in pooled format.

### 3.2. Loss-of-Function and Gain-of-Function CRISPR Screens

CRISPR screens can be divided into three categories by mechanism of action: CRISPRi, CRISPRa, and CRISPR knockout screens.

CRISPR-ko and CRISPRi are loss-of-function approaches. CRISPR-ko screens are based on classical CRISPR/Cas9 systems, which result in DSBs and indel mutations, or on cytidine base editors, which convert codons into stop codons, leading to production of truncated, non-functional proteins (CRISPR-STOP or iSTOP approaches) [28,29]. In addition to gene knockouts, CRISPR base editors can be used to screen gene isoforms formed by single-nucleotide mutations in genetic disorders and cancers [30,31,32]. The main limitation of CRISPR-ko screens is potential arbitrary disruption of genes important for cell viability, which can lead to cell death, loss of sgRNA hits, and misinterpretation of CRISPR-ko screening results. If gene function is not completely disrupted, a truncated protein with altered functions may be produced, also compromising CRISPR-ko results. Cas9-induced DSBs can non-specifically induce genotoxicity, loss of chromosomes, and genome rearrangements, which may undermine the validity of CRISPR-ko screens. In this respect, CRISPR-ko screens using base editors are more reliable, as these enzymes generate low rates of indel mutations and low genotoxicity [33].

In CRISPRi screens, dCas9 is fused to gene repressors (KRAB, KRAB-MeCP2, DNMT3A-3L-dCas9-KRAB (CRISPR-off)), which inhibit target gene activity. Unlike CRISPR-ko, CRISPRi markedly suppresses target gene transcription by epigenetic mechanisms, eliminating the risk of producing truncated proteins and genotoxicity. Additionally, RNA interference, the canonical method for loss-of-function screens, results in less efficient gene knock-down and typically has higher rates of non-specific effects than CRISPRi [34,35].

CRISPRa screening is a gain-of-function method using dCas proteins fused to gene activation domains (VPR [16], SunTag [36], SAM [18]) that can endogenously induce target gene expression [37]. CRISPR screens have several advantages over cDNA libraries, which overexpress specific gene isoforms from robust promoters. Gene activation by CRISPRa allows more physiologic gene levels, rather than the vast overexpression seen using cDNA libraries. Every gene can be activated, in contrast to cDNA libraries, in which the size of the gene and impairment of viral vector production may be a challenge. Finally, CRISPR enables robust activation of all or many isoforms of the same gene. However, CRISPRa/CRISPRi can potentially affect bispecific promoters, thus altering expression of off-target genes by a single gRNA. Efficacy of different gRNAs targeting single genes is also highly variable, resulting in bias in pooled CRISPR screens [38,39].

### 3.3. Classical and Combinatorial CRISPR Screening

In classical CRISPR screens, thousands of gRNAs are introduced into cells with one gRNA per cell. However, certain situations require testing the effects of a combination of genes in one cell. To investigate potential gene interactions and discover new drug targets, CRISPR screens can be performed in combinatorial format, with several genes altered in a single cell. DeWeirdt et al. optimized Cas12a (previously known as Cpf1) nuclease from *Acidaminococcus* sp. Cas12a (AsCas12a) for pooled screens [40]. With Cas12a, several gRNAs can be transcribed by RNA polymerase II as a single polyadenylated transcript, then processed by Cas12a into individual gRNAs [41]. The authors validated the optimized AsCas12a system in performing dual or triple knockout screens [40]. Alternately, combinatorial screening (up to three knockouts per cell) can be done using classical Cas9 with barcoded gRNAs. These barcodes are unique for each assembled combination of gRNAs and thus can be analyzed by NGS. All gRNAs are encoded by a single lentiviral vector. This approach is compatible with dCas9-based CRISPR screening systems [42].

## 4. Workflow of CRISPR Pooled Screening

### 4.1. Design of gRNAs for CRISPR Screens

**Figure 1:**
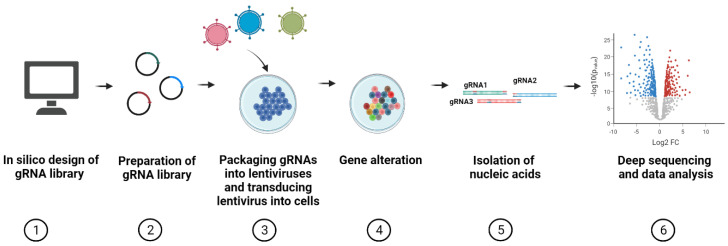
General workflow of CRISPR screens. CRISPR screening protocols include 6 main steps: (1) in silico design of gRNA libraries; (2) cloning and validation of gRNA library; (3) packaging gRNAs into lentiviruses and transduction of lentiviruses into Cas-expressing cells; (4) alteration of genes in experimental conditions; (5) isolation of nucleic acids; and (6) deep sequencing of barcoded gRNAs and data analysis. Note: if using pre-made gRNA libraries, the first two steps of the protocol are omitted.

The main steps of a generalized CRISPR screening protocol are provided in Figure 1. The first step in performing a CRISPR screen is designing the gRNA libraries. Several issues must be considered here. First, gRNA efficacy can vary widely [43] depending on nucleotide sequence, preference of cytosine residues [44], melting temperature [45,46], purine content [47], PAM-distal GC content [48], cell model used, and other features. Several bioinformatics algorithms have been developed to predict gRNA efficiency and specificity. Today, algorithms for calculating gRNA efficiency and specificity are integrated into convenient online tools used for different CRISPR applications, including CHOPCHOP [49], E-CRISPR [50], CRISPOR [51], CRISPRscan [47], CCTop [52], GT-scan [53], GuideScan [54], MultiGuideScan [55], CRIPSRDo [56], and Wu-CRISPR [57]. Most web-based tools allow selection of preferable algorithms for on-target and off-target score estimation. Advantages and limitations of CRISPR gRNA design tools have been reviewed previously [58,59,60].

Epigenetic modification can influence gRNA efficacy. Typically, the most effective gRNAs are located in nucleosome-free sites with open chromatin [60,61]. When designing gRNAs for CRISPRi/CRISPRa, the position of gRNA in the region of the gene transcription start site (TSS) plays a crucial role [43]. Highest gRNA efficacy for CRISPRi is observed for positions located from +50 to +100 nucleotides from the TSS [37], whereas the best window for CRISPRa gRNAs is −150 to −75 nucleotides upstream of the TSS [62,63]. A plethora of studies demonstrated the importance of exhaustive in silico gRNA design for generating valid CRISPR screening results. Several online tools also consider epigenetic features of the target site in gRNA design (Wu-CRISPR [57], Azimuth [46], CRISPRpred [64], TSAM [65], uCRISPR [66]).

Designing gRNAs for base editors can be complicated, as additional factors should be considered during selection. The PAM sequence should be located near the target sequence to introduce the desired mutation or create a stop codon. The editing window of most base editors is narrow (usually 13–17 nucleotides from PAM) and depends on the base editor used [21]. For iSTOP technology, a special database was created containing gRNA sequences that enable knockout of 97–99% of genes in eight eukaryotic organisms. These gRNAs can potentially be used for designing a genome-wide library [28]. gRNAs for custom libraries can be also designed with the convenient web tool BE-Designer, which allows gRNA selection for commonly used base editors [67].

Finally, using one gRNA to target a gene may be insufficient. The common approach is to design at least 4–10 gRNAs targeting a single gene to obtain consistent results between gRNAs and provide high statistical power [68]. However, as the number of gRNAs in a library increases, the screening approach requires larger numbers of cells and increased depth of NGS coverage [46]. Depending on the investigation’s purposes, a library can be designed to alter all genes in the genome (genome-wide format) or to study a single biological pathway.

### 4.2. Available sgRNA Libraries for CRISPR Screens

Pre-made gRNA libraries are publicly available through AddGene. Alternatively, custom sets of gRNAs can be created for different purposes. gRNA libraries can be designed for modulating a specific cellular pathway or the entire genome. Genome-wide libraries provide the most comprehensive screening data but contain a large number of gRNAs (usually >100,000) and thus require huge numbers of cells and very high sequencing depth to reveal screening hits. The most commonly used genome-wide libraries are GeCKO/GeCKOv2 [69,70], Toronto [71], Brunello (CRISPR-ko) [63], Dolcetto [63], CRISPRi-v2 (CRISPRi) [61], Calabrese [63], Mini-Human [72], Gattinara [73], and CRISPRa-v2 (CRISPRa) [61]. These libraries target over 18,000 genes with 4–10 gRNA per gene. GeCKOv2 contains 1864 gRNA sequences targeting miRNA-encoding sequences involved in transcriptional regulation [70]. An important feature of the Gattinara [73] genome-wide library is its small size (~40,000 gRNAs), which allows modulation of nearly 20,000 sequences with two highly effective gRNAs per gene [73]. The recently described BARBEKO library is intended for CRISPR-ko screening using the AncBE4max cytidine base editor that introduces stop codons, disrupts start codons, and introduces splice sites into target genes [74].

Pathway-specific libraries contain fewer gRNAs owing to the limited spectrum of targeted genes, so screening specific pathways is less expensive than using the genome-wide format. Genes that can be targeted with existing libraries for virological research include interferon-stimulated genes (ISGs) [75], cell surface proteins and receptors [76], and ubiquitination/deubiquitination complex genes [77]. The most common human genome-wide or pathway-specific libraries are available for purchase through AddGene (Table 1).

### 4.3. Construction of Cas9- and gRNA-Encoding Vectors

Once gRNAs are designed in silico, gRNA-encoding oligonucleotides are synthesized in microarray format and cloned into vector backbones. The cloned gRNA library is amplified in bacteria and packaged into lentiviral particles. Purified viral particles are titrated to determine the required multiplicity of infection (MOI) [78]. Since these vectors contain an antibiotic resistance gene, titration can be performed by comparing cell numbers before and after antibiotic selection [78]. Viral titers can also be determined via ELISA against the p24 capsid protein, real-time PCR detecting vector insert, fluorescent titration with flow cytometry, and other methods.

Cas9 protein can be expressed by the same vector that encodes the gRNA, or a cell line stably expressing Cas/dCas protein can be established first. Using the same vector for Cas and gRNA is simpler, but the additional Cas-expressing frame greatly increases vector size and diminishes the titer of produced lentivirus. Expressing Cas and gRNA separately reduces the probability of recombination during plasmid amplification, and a virus produced from such a vector can be manufactured with higher titers. If screening assumes the use of a single cell line, generating Cas/dCas-expressing clones is recommended [79].

### 4.4. Lentiviral Transduction

Once lentiviral constructs are made and titrated, target cells are transduced with these lentiviruses at MOI ~0.3–0.4 to obtain cells expressing 0 to 1 gRNA each. Transduced cells are selected by FACS or by treating with antibiotics and cultured for 7–14 days to ensure modulation of the target genes and accumulation of cell mass for subsequent analysis. The approximate number of cells at the end of this stage is calculated according to library size and should exceed 200 cells per gRNA [78].

### 4.5. Selection

**Figure 2:**
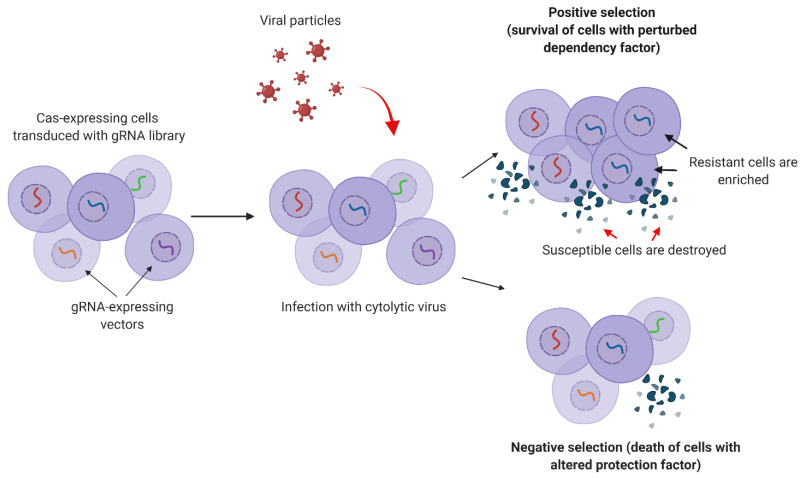
Positive and negative selection of cells for CRISPR screening. Cas-producing cells are transduced with the lentiviral gRNA library. Next, cells are infected with a cytolytic virus and then undergo positive selection (resistant cells are enriched while susceptible cells die) or negative selection (cells with altered antiviral genes die).

Next, cells are selected based on specific markers or phenotypes via negative or positive selection (Figure 2). In negative selection, cells with the desired phenotype are depleted from the cell population. For example, cells can be treated with an anti-tumor drug, so gRNAs that inhibit a drug resistance gene induce cell death. Cells are harvested at different time points after transduction to determine which gRNAs are depleted from the cell population. During negative selection, each gRNA can be depleted from the cell population only to the extent that it was presented in the initial library. If a library consists of 100,000 gRNAs, each gRNA can only be depleted by 0–1 per 100,000 cells, so the reduction in the overall cell pool is negligible. This limitation can be diminished by using fewer gRNAs in a library [43]. Another limitation of negative selection is cell depletion that occurs over time independent of gene modification.

In positive selection, cells with the desired phenotype are enriched in a cell population while other cells are depleted. For example, inhibiting a gene responsible for susceptibility to a virus will allow cells to survive infection in culture. In positive selection, cells with a single modification can in principle be enriched to 100% of the final population, so the effect of a specific gRNA is much more evident than in negative selection.

### 4.6. Sequencing

After selection, genomic DNA of selected cells and control cells is extracted; gRNA-encoding integrations are amplified and ligated with NGS adapters. Amplicons from selected cells and control cells are sequenced with sufficient depth to define the relative abundance of each gRNA before and after selection [62].

### 4.7. Data Analysis

The final step, data analysis, can be performed with developed algorithms (MAGeCK RRA [80], MAGeCK-MLE [81], BAGEL [82], CERES [83], CRISPhieRmix [84], JACKS [85], etc.). According to a study by Bodapati et al. [39] comparing different algorithms, MAGeCK RRA performs best in most applications. When gRNA efficiency varies greatly for a single gene (often observed in CRISPRi/CRISPRa screens), CRISPhieRmix should be used, as it considers such variability. For screens that use several cell lines, common hit genes and cell-specific hit genes can be determined with MAGeCK-MLE, JACKS, and CERES [39]. The commonly used free MAGeCK algorithm has a video tutorial that allows even inexperienced users to utilize it and provides demo data sets for performing test analysis [86]. Significant fold-changes for several gRNAs targeting the same gene have higher statistical power than a single gRNA hit per gene.

The results of the screen should be further validated to exclude false-positive hits and screening bias. For validation, gRNAs for screening hit genes are cloned into individual vectors and used to reproduce the data in separate experiments. The change in target gene expression should be approved by investigating target protein levels.

A comprehensive, step-by-step protocol for different types of CRISPR screening, including detailed troubleshooting, was provided by Joung et al. [87].

## 5. CRISPR Screens for Studying Viral Infections

Viruses affect billions of people worldwide, but specific treatments are not available for most of these infections. Viruses can cause local outbreaks, epidemics, and seasonal brief infections, and many viral agents also cause chronic infections, in which the virus can persist in the body for years, damaging specific tissues, organs, or organ systems. Studying emerging, re-emerging, and persistent viruses and their interactions with host cells is crucial for understanding their biology and host response, identifying novel therapeutic targets, and determining pathways of innate immunity. CRISPR screens provide a great advantage for high-throughput analysis of viral agents and host factors for these purposes. Table 2 summarizes the results of CRISPR screen analyses for different viral infections.

### 5.1. CRISPR Screens for Studying Virus–Host Interactions

#### 5.1.1. Virus Entry and Transmission

Genome-wide screens are often used for basic research in virology and for identifying factors responsible for virus entry into cells and transmission of infection. Hepatitis A virus (HAV) host dependency factors were first investigated using CRISPR screening by Das et al. in 2020 [103]. The study found that components of the ganglioside synthetic pathway are important for HAV entry into cells, and that viral capsids are not uncoated inside ganglioside-deficient cells.

Wei et al. developed another custom gRNA library to analyze severe acute respiratory syndrome coronavirus 2 (SARS-CoV-2) using a CRISPR screen approach. The most prominent hits included high-mobility group protein B1 (HMGB1), which upregulates expression of SARS-CoV-2 entry receptor ACE2. Disrupting the HMGB1 gene in susceptible cells reduced the abundance of active chromatin modifications at the regulatory elements of the ACE2 promoter [94].

In 2017, Park et al. [34] performed a genome-wide CRISPR-ko screen to reveal the factors required for human immunodeficiency virus (HIV) entry and replication. CRISPR screens both confirmed the role of previously described pro-HIV factors like CD4 and CCR5, proving the validity of the screen, and identified three additional factors involved in HIV replication: tyrosylprotein sulfotransferase 2 (TPST2), solute carrier family 35 member B2 (SLC35B2), and activated leukocyte cell adhesion molecule (ALCAM). TPST2 was found to contribute to CCR sulfation, required for HIV–CCR5 interaction. SCL35B2 was shown to be involved in the transportation of 3′-phosphoadenosine-5′-phosphosulfate, a donor of sulfate groups. Depleting SCL35B2 resulted in deficient CCR5 sulfation and impaired HIV entry. Additionally, ALCAM deficiency diminished cell-to-cell transmission of HIV.

Several CRISPR screen studies were performed to investigate human cytomegalovirus (HCMV) host dependency factors. Wu et al. revealed PDGFRα as a top screening hit required for trimer-mediated HCMV entry, as well as for cell-to-cell spread of trimer-only HCMV. Pentamer-containing viruses still infected the PDGFRA-deficient cell, albeit with lower efficiency [109]. A different study demonstrated that the sensitivity of epithelial cells to infection with pentamer HCMV is mediated by OR14I1 protein. PDGFRA and OR14I1 factors were shown to serve as non-redundant co-receptors for HCMV pentameric complex [108].

#### 5.1.2. Viral Replication

Hoffman et al. used custom library CRISPR screens to study SARS-CoV-2 interactome with infected cells [123]. Along with SARS-CoV-2, the authors utilized the CRISPR screen to search for pan-coronavirus factors required for replication in models of HCoV-NL63, HCoV-229E, and HCoV-OC43 infection. Sterol regulatory element-binding protein cleavage-activating protein (SCAP) was identified as the host factor important for replication of all four coronaviruses. In healthy cells, SCAP regulates lipid and cholesterol homeostasis by sequestering sterol regulatory element-binding proteins in the endoplasmic reticulum. SCAP may promote coronavirus infection by hijacking SREBPs-dependent transport and/or by potentiating viral interactions with cell membranes when cholesterol content is increased [124].

#### 5.1.3. Viral Protein Stability

Lin et al. studied virus–host interaction factors of dengue virus (DENV) infection using a CRISPR-ko screen, showing the importance of host factor MAGT1 [98]. Two subunits of DPMS complex (DMP1 and DMP3) were also demonstrated to play a role in DENV infection by regulating DENV replication and enhancing viral structural glycoprotein stability [97].

### 5.2. CRISPR Screens for Identifying New Antiviral Targets

The search for new therapeutic targets for treating viral diseases is an important branch of drug discovery. CRISPR screens make it possible to search for viral host factors important for viral replication and maintenance in a high-throughput manner.

#### 5.2.1. Latency

In 2018, Jin et al. used CRISPR screening to identify genes maintaining HIV latency, a state in which the integrated provirus is not transcribed [92]. Latency allows HIV persistence and prevents immune-mediated clearance of infected cells. Reactivating the latent provirus along with anti-retroviral therapy is a shock-and-kill strategy that can potentially destroy latent reservoirs and eliminate the virus. Thus, identifying new targets for reactivating HIV is important for developing novel therapeutic approaches. CRISPR-ko screening revealed that TSC1 and DEPDC5 maintained suppression of AKT-mTORC1 signaling, thus promoting HIV latency. Inactivating these genes induced activity of the AKT–mTORC1 axis and stimulated HIV replication [92]. In another study, Li et al. (2020) used genome-wide CRISPRi screening to demonstrate that inhibiting FTSJ3, TMEM178A, and NICN1 genes stimulated RNA polymerase II-mediated transcription of HIV, promoting its latency. Further immunoprecipitation experiments showed that depleting TMEM178A and NICN1 increased polymerase II signaling on HIV’s long terminal repeat (LTR) regulatory elements and envelope region. Thus, these two genes affect both initiation and elongation of HIV-1 transcription. Depleting FTSJ3 and INTS2 increased polymerase II signaling in the envelope region but not in LTR, suggesting a role for these proteins in elongation [88]. A CRISPR-ko screen by Yang et al. revealed phosphatidylethanolamine-binding protein 1 (PEBP1) as a gene promoting HIV latency via dephosphorylation-mediated inactivation of MAPK and NF-kB signaling pathways [89]. KRAB-containing zinc-finger protein ZNF304 was recently identified in a CRISPR screen as a silencer of the HIV promoter, and, thus, as a factor promoting HIV latency [90]. Rathore et al. used a CRISPR-ko screen to discover additional genes involved in HIV latency, including IWS1, POLE3, POLR1B, PSMD1, TGM2, UCH37, CYLD, A 20, OTULIN, USP5, and USP14. Pharmacological inhibition of some factors, such as UCH37 and USP14, reversed HIV latency and induced provirus reactivation [91]. Overall, these studies provide valuable information about potential drug targets to reactivate HIV replication and develop novel antiviral approaches for HIV patients.

#### 5.2.2. Entry

Schneider et al. conducted a genome-wide CRISPR-ko screen in models of natural SARS-CoV-2 infection at temperatures observed in the upper (33 °C) and lower (37 °C) respiratory tract [93]. One of the top screening hits was ACE2, a previously established receptor for SARS-CoV-2. In primary analysis, 84 (37 °C) and 99 (33 °C) genes were shown to facilitate SARS-CoV-2 infection. Among these, transmembrane protein 41B (TMEM41B) was one of the most important host factors required for infection by SARS-CoV-2 and other seasonal coronaviruses (HCoV-OC43, HCoV-NL63, and HCoV-229E). The role of TMEM41B in the SARS-CoV-2 life cycle is not unique to coronaviruses, as TMEM41B is also important for infection by flaviviruses (for example, Zika virus (ZIKV)) [93].

In 2019, a wide-genome CRISPR screen was implemented by Flint et al. in a search for host factors required for Ebola virus (EBOV) replication. GNPTAB, which encodes N-acetylglucosamine-1-phosphate transferase, was identified. The authors showed that EBOV infection was inhibited in the absence of GNPTAB, concluding that disrupting GNTAB function can be a strategy for treating EBOV infection [114].

#### 5.2.3. Protein Translation

Hepatitis B virus (HBV) causes one of the most widespread viral infections, inducing cirrhosis and hepatocellular carcinoma. Currently, therapy can suppress viral replication and reduces the risks of severe outcomes. In 2019, Hyrina et al. performed a CRISPR screen to identify host factors required for viral replication and production of HBsAg, an important biomarker of viral replication [104]. Top hits included zinc-finger CCHC-type containing 14 (ZCCHC14) protein, which was shown to stabilize HBV S-mRNA and promote HBsAg expression. Additionally, ~60 genes were shown to influence HBsAg levels. All identified genes are potential therapeutic targets for managing chronic HBV infection. Clearance of HBsAg in patients with chronic HBV infection is a major therapeutic target that may help to provide durable control over viral replication and substantially reduce the risk of liver cirrhosis and hepatocellular carcinoma [125].

CRISPR screen technology is suitable for finding new therapeutic targets to treat viral infections. The most potent target factors can be blocked by small-molecule inhibitors, monoclonal antibodies, or RNA interference drugs for antiviral therapy. Additionally, factors with pronounced antiviral effects can be upregulated using CRISPRa or other strategies for therapeutic purposes [126,127,128].

### 5.3. Immunity Studies

Richardson et al. used CRISPR screening to identify genes that regulate interferon (IFN) response to flavivirus infection. Cells were treated with a high dose of IFN-α before running a CRISPR screen to identify factors that make cells susceptible to infection. Members of IFN-α signaling pathway IFNAR1, IFNAR2, IRF9, and ISG effector gene IFI6 were revealed as factors with the highest antiviral activity. The authors showed that IFI16 prevents formation of virus-induced invaginations in the endoplasmic reticulum membrane and impairs viral replication [118].

OhAinle et al. created an ISG targeting library to study HIV infection, showing that a set of ISGs (MxB, TRIM5α, IFITM1, and tetherin) effectively suppressed HIV-1 infection, although, individually, these factors had modest antiviral effects [129]. These findings indicated that instead of using single genes as candidate drug targets, concerted activation of multiple genes is sometimes required to achieve effective viral suppression. In another CRISPR-ko screen targeting 1906 human ISGs with 8 gRNAs per gene, Roesch et al. (2018) identified IFITM factors as potent inhibitors of lentiviral particle delivery. IFITM1/3 showed an evident antiviral effect in a model of VSV-g pseudotyped viral-like particles encoding the HIV Vpx gene [75].

Using a CRISPR-ko screen, Li et al. (2019) showed that ZIKV replication relies on many host factors involved in heparin sulfation, endocytosis, and endoplasmic reticulum protein processing. Also, ZIKV actively suppresses the IFN pathway (ISG15 and others), and knocking out ISG15 protected human neural progenitor cells from ZIKV [102]. In 2019, Dukhovny [101] identified that other ISGs, such as IFNL2 and IFI6, can rescue cells from ZIKV infection. Another study using a CRISPR-ko screen [100] identified integrin αvβ5 as a potential therapeutic target for ZIKV; αvβ5 directly interacts with ZIKV and provides the molecular basis for ZIKV internalization.

Using a CRISPR-ko screen, Chia et al. identified genes restricting influenza A virus (IAV) after IFN type I treatment. Along with key components of IFN signaling, replication termination factor 2 (RTF2), a new factor suppressing IAV transcription and upregulation of antiviral networks was identified [130] in this screen.

To conclude, genome-wide CRISPR screens are powerful tools for studying virus–host interactions and for identifying potential therapeutic targets for treating viral infections. Differences in data sets from independent CRISPR screens for the same viral infections can be explained by differences in experimental setups, models of infection used, or types of CRISPR screening. However, most screening hits typically overlap between studies. To increase the statistical power of studies, top hit genes should be validated in different models of viral infection.

## 6. Conclusions

In this paper, we describe the main types, principles, and steps of CRISPR screens. Since their adaptation for genome-wide screening, CRISPR tools have contributed greatly to fundamental and translational studies. Many types of Cas proteins and sgRNAs have been used to develop gain-of-function CRISPRa and loss-of-function CRISPRi tools, as well as nuclease-based and base editor-mediated approaches. Additionally, CRISPR screening approaches have become powerful tools for studying viral infections, identifying host dependency factors and novel drug targets. Given the recent breakthroughs in artificial intelligence and modeling of protein 3D structures, designing new small molecules has become much more efficient. Combining these approaches will advance drug discovery in the coming years. Combinatorial CRISPR screens provide the opportunity to analyze complex biological processes and define the effective combinations of antiviral factors with initially low or mediocre antiviral activity. This will be important for optimizing existing therapeutic approaches and developing new ones.

## Figures and Tables

**Table 1:** Currently available pre-made CRISPR libraries.

Type of Screen	Library Name	AddGene Catalog Number	Genes Targeted	gRNAs Per Gene	Total gRNAs
**Genome-Wide Libraries**					
Ko	GeCKO v2	1000000048 and 1000000049	19,052	6	123,411
Ko	Toronto v3	90294 and 125517	18,053	4	70,948
Ko	Brunello	73179 and 73178	19,114	4	76,441
Ko	Gattinara	136986	19,993	2	40,964
Ko	Mini-Human AsCas12a-Based Library	130630	16,997	3–4	17,032 arrays (3–4 gRNAs per array)
Ko	BARBEKO (for screening with a base editor)	174163	17,501	3	53,502
CRISPRi	Dolcetto	1000000114	18,901 (Set A); 18,899 (Set B)	3–6	57,050 (Set A); 57,011 (Set B)
CRISPRi	CRISPRi-v2	83969 1000000090	18,905	5–10	104,535 209,070
CRISPRa	Calabrese	1000000111	18,885 (Set A); 18,843 (Set B)	3–6	56,762 (Set A); 56,476 (Set B)
CRISPRa	CRISPRa-v2	83978 1000000091	18,915	5 or 10	104,540 or 209,080
CRISPRa	SAM Library (3-plasmid system)	1000000057 and 1000000074	23,430	3	70,290
**Pathway-Specific Libraries**					
Ko	Human Interferon-Stimulated Gene CRISPR Knockout Library	125753	1902	8	15,416
Ko	Bison sgRNA Library (ubiquitination and deubiquitination genes)	169942	713	4	2852
Ko	Li Human UBDUB CRISPR Knockout Library (ubiquitination and deubiquitination genes)	171531	1500	~6	9274
CRISPRa	Wright Human Membrane Protein Activation Library (surface proteins)	113345	6213	7–14	58,570

**Table 2:** CRISPR screens for studying viral infections.

Virus	Screen Type	gRNA Library	Cells	Top Screening Hits	Ref.
HIV	CRISPR-ko	Custom library of 187,536 gRNAs targeting 18,543 genes	GXR cell line	TPST2, SLC35B2, ALCAM, CCR5, CD4	[34]
	CRISPRi	CRISPRi-v2	Jurkat	TMEM178A, FTSJ3, INTS2, INTS5, INTS8, NICN1	[88]
	CRISPR-ko	GeCKO	C11 cell line	PEPB1, BRD2, BRD4	[89]
	CRISPR-ko	GeCKO	Jurkat	ZNF304	[90]
	CRISPR-ko	GeCKO v2	Jurkat (J-Lat 10.6 cell line)	52 genes including IWS1, POLE3, POLR1B, PSMD1, TGM2	[91]
	CRISPR-ko	GeCKO v2	Jurkat (HIV-1 latent infection cell line [C11])	TCS1, DEPDC5, SUV39H1, SPATA6L, NFKB2, and other genes	[92]
Coronaviruses (SARS-CoV-2, MERS)	CRISPR-ko	Brunello	Huh-7.5	146 (37 °C) and 171 (33 °C) genes, including TMEM41B for all of investigated viruses	[93]
	CRISPR-ko	Custom library of 83,963 gRNAs	Vero-E6	Genes of SWI/SNF complex, ACE2, DPP4, CTSL, PCBD1, KMT2D, SMAD3, HMGB1, and others	[94]
	CRISPR-ko	GeCKO v2	A549^ACE2^	RAB7A, CCDC22, VPS35, ACE2, CTSL, and others	[95]
	CRISPR-ko	GeCKO v2	Huh7.5.1	TMEM106B and other genes	[96]
Dengue virus (DENV)	CRISPR-ko	GeCKO v2	Haploid HAP1 cells	17 genes including DPM1 and DPM3	[97]
	CRISPR-ko	GeCKOv2	Huh7.5.1	STT3A, STT3B, DC2, MAGT, RPN2, OST4	[98]
Zika virus (ZIKV), yellow fever virus	CRISPR-ko	GeCKO	HAP1	TMEM41B and VMP1 (overlap between the two viruses)	[99]
ZIKV	CRISPR-ko	Brunello	TS576	CENPH, ITGB5, MYLPH, HOMER1, BAALC, GABBR2, EPHA10, PTNP2, GCNT7, TRAM1, TMEM41B	[100]
	CRISPRa	LentiSAMv2	Huh7	IFI6, IFNL2, ISG20, HELZ2	[101]
	CRISPR-ko	LentiCRISPRv1; custom library	Human neural progenitors cells	TM9SF2, ATP6V1C1, ATP6V1F, SSR2, SSR3, EMC2, EMC6, C3orf58, ISG15, SOCS3, STAT3	[102]
Hepatitis A virus (HAV)	CRISPR-ko	Brunello	HeLa	39 genes including UGCG, GALE, and SLC35A2	[103]
Hepatis B virus (HBV)	CRISPR-ko	Custom library of 19,050 genes, with 5 gRNAs/gene	HepG2	22 pro-HBsAg genes, including ZCCHC14, NXT1, and ENY2; 38 anti-HBsAg genes, including DCAF7, UBE2J1, RNF139, and UBE2J2	[104]
Epstein-Barr virus	CRISPR-ko	AVANA	P3HR-1	MYC, EP300, STAGA, FACT, cohesin subunits	[105]
	CRISPR-ko	Brunello	Lymphoblastoid cell lines (LCL)	TAF family proteins, MEF2C	[106]
	CRISPR-ko	Avana	P3HR-1, GM12878	57 genes for P3HR1 and 87 genes for GM12878	[107]
Human cytomegalovirus (HCMV)	CRISPR-ko	GeCKO v2	ARPE-19; HEL fibroblasts	OR14I1, PDGFRA	[108]
	CRISPR-ko	GeCKO v2	Human foreskin fibroblasts (HFF)	PDGFRA	[109]
Influenza A virus (IAV)	CRISPR-ko	AVANA-4	A549	WDR7, CCDC115, TMEM199, CMTR1	[110]
	CRISPR-ko	GeCKO	A549	SLC35A1 and other genes	[111]
Adeno-associated virus	CRISPR-ko	GeCKO	Huh7	GPR108, NEU1, GCNT4, CTSA	[112]
	CRISPR-ko	GeCKO v2	Huh7	Crb3, CLDN15	[113]
Ebola virus (EBOV)	CRISPR-ko	GeCKO v2	Huh7.5.1	GNPTAB, NPC1, SPNS1, SLC30A1, HOPS complex, UVRAG	[114]
Enteroviruses (RV-C15 and non-polio EV-D68)	CRISPR-ko	GeCKO v2	H1-HeLa cells	SETD3, CSDE1, PLA2G16	[115]
Severe fever with thrombocytopenia syndrome virus	CRISPR-ko	GeCKO v2	HeLa	SNX11	[116]
Norovirus restriction factors	CRISPRa	Calabrese	Hela	TRIM7, PITX1, HOXC11, DDX60, MX1, PLSCR1	[117]
Flaviviruses	CRISPR-ko	Brunello	Huh7.5	IFI6, STAT2, IRF9	[118]
	CRISPR-ko	GeCKO v2	293T	STT3A, SEC63, SPCS1, SPCS3	[119]
West Nile virus	CRISPR-ko	Custom library of 77,406 gRNAs covering 20,121 genes	293FT	EMC2, EMC3, SEL1L, DERL2, UBE2G2, UBE2J1, HRD1	[120]
Arthritogenic alphaviruses (chikungunya, Ross River, Mayaro, O’nyong nyong)	CRISPR-ko	GeCKO v2	3T3	MXRA8	[121]
Rotavirus	CRISPR-ko	GeCKO	H1-Hela	SLC35A1, GNE, CMAS, UGCG, FA2H, LATS2, STAG2	[122]
